# Oxidative Stress Biomarkers in Urine of Metal Carpentry Workers Can Be Diagnostic for Occupational Exposure to Low Level of Welding Fumes from Associated Metals

**DOI:** 10.3390/cancers13133167

**Published:** 2021-06-24

**Authors:** Flavia Buonaurio, Maria Luisa Astolfi, Daniela Pigini, Giovanna Tranfo, Silvia Canepari, Antonio Pietroiusti, Iacopo D’Alessandro, Renata Sisto

**Affiliations:** 1 Department of Chemistry, Sapienza University, 00185 Rome, Italy; flavia.buonaurio@uniroma1.it (F.B.); marialuisa.astolfi@uniroma1.it (M.L.A.); 2 Department of Occupational Medicine, Epidemiology and Hygiene, INAIL Research Center, Monte Porzio Catone, 00078 Rome, Italy; d.pigini@inail.it (D.P.); r.sisto@inail.it (R.S.); 3 Department of Environmental Biology, Sapienza University, 00185 Rome, Italy; silvia.canepari@uniroma1.it; 4 Department of Biomedicine and Prevention, University of Tor Vergata, 00133 Rome, Italy; pietroiu@uniroma2.it (A.P.); iacopodalessandro@libero.it (I.D.)

**Keywords:** occupational exposure, toxic metals, mixtures, human biomonitoring, effect biomarkers, oxidative stress

## Abstract

**Simple Summary:**

The main objective of this study is to investigate the association between biomarkers of human exposure to toxic elements and effect biomarkers of oxidative stress. The concentrations of eight toxic elements in the urine of metal carpentry workers were found to be higher than in those of controls. They were also associated with higher values of RNA and protein oxidative stress biomarkers, showing the presence of an oxidative stress condition linked to the occupational exposure, probably to the mixture of those elements that can accumulate in the body’s tissues. In particular, the determination of urinary 8-oxo-7,8-dihydroguanosine results are valuable support to identify those workers that can be defined as “occupationally exposed” to the used chemicals, among whom appropriate prevention measures and medical surveillance must be applied.

**Abstract:**

Urinary concentrations of 16 different exposure biomarkers to metals were determined at the beginning and at the end of a working shift on a group of workers in the metal carpentry industry. Five different oxidative stress biomarkers were also measured, such as the oxidation products of RNA and DNA metabolized and excreted in the urine. The results of workers exposed to metals were compared to those of a control group. The metal concentrations found in these workers were well below the occupational exposure limit values and exceeded the mean concentrations of the same metals in the urine of the control group by a factor of four at maximum. Barium (Ba), mercury (Hg), lead (Pb) and strontium (Sr) were correlated with the RNA oxidative stress biomarker, 8-oxo-7, 8-dihydroguanosine (8-oxoGuo), which was found able to discriminate exposed workers from controls with a high level of specificity and sensitivity. The power of this early diagnostic technique was assessed by means of the ROC curve. Ba, rubidium (Rb), Sr, tellurium (Te), and vanadium (V) were correlated with the level of the protein oxidation biomarker 3-Nitrotyrosine (3-NO_2_Tyr), and Ba, beryllium (Be), copper (Cu), and Rb with 5-methylcytidine (5-MeCyt), an epigenetic marker of RNA damage. These effect biomarkers can help in identifying those workers that can be defined as “occupationally exposed” even at low exposure levels, and they can provide information about the impact that such doses have on their health.

## 1. Introduction

Metal carpentry is a typical task carried out at industrial sites, often connected with the maintenance of mechanical and electrical/electronic systems. Workers are involved in a wide range of activities that include disassembling and reassembling equipment, mechanical processing with machine tools, and welding and cutting metallic parts, which can lead to occupational exposure to particles and fumes containing metals and metal oxides.

Welding fumes include several toxic substances, such as chromium (Cr), nickel (Ni), cadmium (Cd), and lead (Pb), and also oxidized metal particles of respirable size [1]. Welding fumes have been considered Group 1 human carcinogens by the International Agency for Research on Cancer (IARC) since 2017 [2]. Electronic components can include a variety of harmful metals to which workers may be exposed, including aluminum (Al), antimony (Sb), arsenic (As), beryllium (Be), cadmium (Cd), chromium (Cr), cobalt (Co), mercury (Hg), and nickel (Ni) [3].

Human exposure to heavy metals, such as Cr, Ni, Cd, and Pb, and to welding fumes may cause the formation of reactive oxygen species (ROS) [1,4]. Oxidatively generated damage to DNA and RNA plays an important role in cancer development, cardiovascular and neurodegenerative diseases, diabetes, and pulmonary fibrosis [5,6]. Attacks of ROS on DNA and RNA lead to the urinary excretion of 8-oxo-7,8-dihydroguanine (8-oxoGua), 8-oxo-7,8-dihydro-2′-deoxyguanosine (8-oxodGuo), and 8-oxo-7,8-dihydroguanosine (8-oxoGuo). For this reason, these molecules were considered biomarkers of the effect of oxidatively generated damage. While they are always present in human urine samples because of exposure to oxidative stress agents, arising from different sources [7], a dose–response relationship can be used to emphasize the importance of one of them in particular. These oxidation reactions can also affect lipids and proteins; the oxidation of tyrosine leads to the excretion of 3-Nitrotyrosine (3-NO_2_Tyr), one of the most important protein oxidation biomarkers [8]. The methylation product of cytidine, 5-methylcytidine (5-MeCyt), is an epigenetic marker of RNA damage, and reduced levels of this modified nucleoside are associated with various tumors. On the other hand, RNA methylation is essential for accurate and efficient protein translation, and the appearance of 5-MeCyt in RNA affects the stability and activity of RNA molecules as well as their interaction with their specific ligands [9].

Significantly positive correlations were found between the concentrations of Cd, Cr, copper (Cu), Iron (Fe), manganese (Mn), Pb, titanium (Ti), and zirconium (Zr) and those of the oxidative stress biomarkers 8-oxoGua, 8-oxoGuo, 5-MeCyt, and 3-NO_2_Tyr in the urine samples of workers from a production plant responsible for producing a titanium dioxide pigment [10].

The potential adverse effects of long-term exposure to low-dose complex mixtures, close to health-based reference values, are still relatively unknown [11]. Effect biomarkers can provide the missing information, providing a means of preventing the development of occupational diseases. Exposure biomarkers of internal dose are linked to the amount of chemicals absorbed and excreted by the individual in response to the external exposure.

The aim of this study was to identify the biomarkers of effect that correlated best with the specific biomarkers of exposure, in a healthy population of metal carpentry workers. These biomarkers can be used in medical surveillance as early markers of still-reversible adverse health effects.

## 2. Materials and Methods

### 2.1. Sampling Design

The group analyzed in this study included 40 workers, all males, performing welding activities, who provided urine samples before and after their working shift, and 13 male employees of the same company, who provided only their first morning urine sample. Some workers also provided a hair sample that was tested for its Hg content. All samples were collected on the same day, in the middle of the working week. Before providing the samples, all subjects gave their written informed consent to participate in the study.

Workers were equipped with the most appropriate personal protective devices, and assessment of their exposure to chemical agents was carried out by the employer according to Italian legislation. Specific analytical tests were periodically performed by the company for inhalable dusts, using both personal and environmental samplers; for potentially exposed workers, the biomonitoring of blood Pb and urinary Cd was performed, as part of their medical surveillance. All results were compliant with the Italian occupational exposure limits.

The study can be defined a non-interventional/observational study, on the basis of the European Directive 2001/20/EC, for which the approval of an ethics committee is not required [12]; all experiments were conducted according to the Declaration of Helsinki and followed the International Code of Ethics for Occupational Health Professionals [13], published by the International Committee of Occupational Health (ICOH). The information gathered was used as aggregate data referring to the whole group of workers, with no risk of individual identification.

Urine samples were collected by the workers in sterile plastic containers, divided into three different polypropylene screw cap tubes, and then immediately transported refrigerated to the laboratory, where they were stored frozen at −25 °C until analysis.

Oxidative stress biomarkers were determined on the first aliquot of each urine sample, the creatinine concentration on the second one, while the third one was used to measure the element concentrations. The concentrations of the analytes were expressed as the ratio to the creatinine concentration of the same sample, to normalize their values with respect to the variability of urine dilution grade.

Smoking status was defined by means of the urinary concentration of cotinine, one of the metabolites of nicotine. The classification of smokers was based on a cutoff of 100 µg of cotinine/g of creatinine [14].

Determination of smoking status was needed as data from the literature show that smokers have higher levels of urinary Cd [15] and of 8-oxoGua and 3-NO_2_Tyr [16].

Urinary creatinine was determined using the alkaline picrate test with UV/Vis detection at 490 nm [17]: samples having a creatinine concentration higher than 3 g/L or lower than 0.3 g/L were discarded, according to the recommendations of the American conference of Governmental Industrial Hygienists (ACGIH) [18].

### 2.2. Hair Collection

Hair samples were cut and collected in small plastic bags by the occupational physician appointed by the company for the medical surveillance. Due to the small quantity available, they were tested only for their Hg content.

### 2.3. Chemicals and Supplies

The analytical reference standards of 8-oxoGua, 8-oxoGuo, and 8-oxodGuo were provided by Cambridge Isotope Laboratories, Inc. (CIL) (Tewksbury, MA, USA). The isotope-labeled internal standard (^13^ C^15^ N_2_) 8-oxoGua (98%) was provided by Cambridge Isotope Laboratories Inc. (Tewksbury, MA, USA); (^13^ C ^15^ N_2_) 8-oxoGuo (^13^ C ^15^ N_2_) and 8-oxodGuo (^13^ C ^15^ N_2_) were provided by CDN Isotopes Inc. (Pointe-Claire, QC, Canada); 3-NO_2_Tyr was provided by Cayman Chemical Company (Ann Arbor, MI, USA) and 3NO_2_Tyr d3 by Toronto Research Chemicals (Toronto, ON, Canada). Cotinine (>99.5%) and Cotinine d3 (99%) were supplied by Sigma-Aldrich (Milan, Italy). Glacial acetic acid, 30% NH_3_, dimethyl sulfoxide, sodium hydroxide solution (50–52% in water), CHROMASOLV^®^ gradient grade 99.9% methanol and acetonitrile for HPLC/MS 99.9%, and low benzene content carbon disulfide were purchased from Sigma-Aldrich (Saint Louis, MO, USA). Purified water was produced by a Milli-Q Plus system (Millipore Milford, MA, USA). Anotop 10LC syringe filter devices (0.2 m pore size, 10 mm diameter) were obtained from Whatman Inc. (Maidstone, UK). Analytical columns Luna C8 100 Å (250 × 4.6 mm, 5 µm) (Phenomenex, Torrance, CA, USA) and Discovery C18 (150 × 4.6 mm, 5 µm) (Supelco Analytical, Bellefonte, PA, USA) were used for the study.

All the elements’ calibration solutions were prepared for inductively coupled plasma mass spectrometry (ICP-MS) using a multi-element standard solution (Ultra Scientific/Agilent Technologies, North Kingstown, RI, USA) and for cold vapor atomic fluorescence spectrometry (CV-AFS) by Hg standard solution (SCP Science, Baie D’Urfé, QC, Canada). Super pure analytical reagents of 67% HNO_3_ and 36% HCl were obtained from Carlo Erba Reagents S.r.l. (Milan, Italy), while 30% H_2_O_2_ was purchased by Merck KGaA (Darmstadt, Germany). For the CV-AFS analysis, 5% HCl was used as a carrier and 0.05% NaBH_4_ (Sigma-Aldrich Chemie GmbH, Buchs, Switzerland) in 0.05% NaOH (98%, anhydrous pellets, Carlo Erba Reagents, Milan, Italy) as reducing agent.

### 2.4. Analytical Determination of Urinary Oxidative Stress Biomarkers

The urine samples were analyzed by liquid chromatography/tandem mass spectrometry (HPLC/MS-MS) composed by an API 4000 triple-quadrupole mass spectrometry detector equipped with a Turbo Ion Spray (TIS) probe (AB Sciex, Framingham, MA, USA) coupled to a Series 200 LC quaternary pump (PerkinElmer, Norwalk, CT, USA).

Detection was carried out in the multiple reaction monitoring mode (MRM) and parameters were optimized for the analytes by the automated infusion quantitative optimization procedure and then refined by flow injection analysis (FIA) using the pure standards.

The concentrations of 8-oxoGua, 8-oxoGuo, 8-oxodGuo, and 3-NO_2_Tyr were determined according to a previously described method [19], with modifications in the sample thawing, dilution solvents, chromatographic column, and mobile phases. Before the analysis, samples were thawed in lukewarm water at around 37 °C, vortexed, and centrifuged at 10,000 × *g* for 5 min; the urine supernatant was added with internal standard and injected into the HPLC-MS/MS system. The isotope-labeled internal standard for 8-oxodGuo is 8-oxodGuo (^13^ C ^15^ N_2_), which is commercially available. The reference standards of the analytes were first dissolved in DMSO, then in methanol, and finally diluted with water. The chromatographic column was a Luna C8 100 Å (Phenomenex, Torrance, CA, USA) (250 × 4.6 mm, 5 µm) and the mobile phase consisted of a gradient of a mixture of CH_3_CN and methanol 9:1 *v/v* and 0.5% acetic acid (all purchased from CARLO ERBA Reagents S.r.l., Cornaredo MI, Italy) in water. 

The same method was also used for 5-methylCytidine (5-MeCyt) (Sigma-Aldrich (Milan, Italy) and cotinine (Sigma-Aldrich (Milan, Italy), but after diluting the sample 1:100, and using a different chromatographic column, a Discovery C18 (150 × 4.6 mm, 5 µm) provided by Merck KGaA, Darmstadt, Germany; Cotinine d3 was used as the internal standard for 5-MeCyt. The precursor/product ionic transitions monitored (positive ion mode) were 168.0 → 140.0 and 171.0 → 143.0 for 8-oxoGua and its internal standard ((^13^ C ^15^ N2) 8-oxoGua), 284.3 → 168.0 and 287.13 → 171.1 for 8-oxodGuo and its internal standard ((^13^ C ^15^ N2) 8-oxodGuo), 300.24 → 168.2 and 303.24 → 171.0 for 8-oxoGuo and its internal standard ((^13^ C^15^ N2) 8-oxoGuo), 226.99 → 181.0 and 229.99 → 184.0 for 3-NO_2_Tyr and its internal standard (3-NO_2_Tyr d3), 257.95 → 126.100; 180.3 → 80.10 was the transition monitored for 5-MeCyt, 177.3 → 80.10 for cotinine, and 180.3 → 80.10 for cotinine-d3 used as internal standard for both 5-MeCyt and cotinine.

The limits of detection (LODs) were 0.50 µg/L for 8-oxoGua, 0.14 µg/L for 8-oxodGuo, 0.70 µg/L for 8-oxoGuo, 1.81 µg/L for 3-NO2Tyr, 0.28 µg/L for 5-MeCyt, and 12.41 µg/L for cotinine.

The limits of quantification (LOQs) were 1.67 µg/L for 8-oxoGua, 0.48 µg/L for 8-oxodGuo, 2.33 µg/L for 8-oxoGuo, 6.03 µg/L for 3-NO2Tyr, 0.94 µg/L for 5-MeCyt, and 41.37 µg/L for cotinine.

The 1.5 version of Analyst^®^ software (AB Sciex, Framingham, MA, USA) was used for instrument control.

### 2.5. Analytical Determination of Urinary Elements

Urinary elements were analyzed by ICP-MS (820-MS; Bruker, Bremen, Germany) equipped with a collision reaction interface (CRI) for Ba, Be, Bi, Cd, Cs, Cu, Fe, Ni, Pb, Rb, Sb, Se, Sr, Te, and V and by CV-AFS (AFS 8220 Titan, FullTech Instruments, Rome, Italy) for Hg, as described previously, with minor modifications [20,21,22]. The parameters and operating conditions of ICP-MS are detailed elsewhere [23]. Briefly, Fe, Se, and V were determined by CRI with He and H_2_ (99.9995% purity, SOL Spa, Monza, Italy) as cell gases. Urine samples were diluted 5-fold with 3% HCl (30%, suprapure, Carlo Erba Reagents S.r.l., Milan, Italy) and 10-fold with 2% HNO3 (67%, suprapure, Carlo Erba Reagents S.r.l. Milan, Italy) in polypropylene tubes (Artiglass s.r.l., Due Carrare, PD, Italy) and filtered before CV-AFS and ICP-MS analysis, respectively. The LODs and LOQs for all the selected elements were, respectively: Ba, 3 and 10 µg/L; Be, 0.03 and 0.09 µg/L; Bi, 0.01 and 0.04 µg/L; Cd, 0.05 and 0.2 µg/L; Cs, 0.01 and 0.04 µg/L; Cu, 0.5 and 2 µg/L; Fe, 2 and 5 µg/L; Hg, 0.03 and 0.1 µg/L; Ni, 0.7 and 2 µg/L; Pb, 0.04 and 0.1 µg/L; Rb, 0.08 and 0.3 µg/L; Sb, 0.01 and 0.05 µg/L; Se, 1 and 3 µg/L; Sr, 0.6 and 2 µg/L; Te, 0.2 and 0.6 µg/L, and V, 0.4 and 1 µg/L.

### 2.6. Analytical Determination of Hair Hg

The Hg determination in hair was carried out using an Advanced Mercury Analyzer (AMA-254, Altec Ltd., Prague, Czech Republic) with O_2_ (99.995% purity; SOL Spa, Monza, Italy) as carrier gas [24]. Briefly, a hair sample (~5 mg) was weighed into the analyzing Ni shuttle and introduced into the instrument without any pre-treatment. Blanks were analyzed periodically to verify that Hg was not carried over between samples. The method detection limit for Hg in hair was 0.002 ng, which corresponds to a concentration of 0.0004 mg/kg (sample hair mass of 5 mg).

## 3. Statistics

Statistical analysis was performed using the IBM SPSS statistics 25 software or R version 3.5.3 (2019-03-11, The R Foundation for Statistical Computing, Vienna, Austria).

All the concentration data were log-transformed as their original distributions were lognormal.

A two-tailed Student *t*-test for independent heteroskedastic variables was used to analyze the differences between the log-transformed urinary concentrations in workers and controls. A coupled *t*-test was used to compare the urinary concentrations of metals at the beginning and the end of the working shift. A *p*-value lower than 0.05 was considered statistically significant.

Two different ANOVA tests for repeated measures (IBM SPSS Statistics 25, IBM Corp., Armonk, NY, USA) were used to verify the differences between exposed subjects, before and after the working shift, BS and ES, respectively, and the controls. In the first test, the difference in the exposure variables, i.e., the 16 metal concentrations, was analyzed. In the second test, the five biomarker concentrations were considered as repeated-measure variables. In both ANOVA tests, a three-level factor, “exposure”, referring to the exposure before and after the working shift, and to the absence of exposure, was tested as the “between subjects” variable.

Centered and scaled data—i.e., for each variable, the difference between the variable value and the grand mean divided by the standard deviation—were analyzed by means of Principal Component Analysis (PCA), with the aim of visualizing the difference between the exposed workers, evaluated at the beginning and the end of the working shift, and the control group (IBM SPSS Statistics 25). PCA was performed both with respect to all the variables, metal concentrations, and effect biomarkers, and with respect to the effect biomarkers separately.

The Receiver Operating Characteristic (ROC) curves were studied for the different biomarkers (R 3.5.3). The ROC curve is a technique measuring the diagnostic power of a dichotomic test, which gives a positive output if a given parameter is above/below a specified threshold. Varying the threshold value as a free parameter, the fraction of samples that are correctly classified (hits) as positive is plotted against the fraction of samples that are incorrectly classified as positive (false alarms). The fraction of hits represents the test sensitivity, 1-*β*, where *β* is the probability of an error of type II. The complement to the fraction of false alarms α (type I errors) represents the specificity of the test (1-α). Varying the threshold level, the fraction of hits, as well as the fraction of false alarms, increases monotonically from zero to 1. In a highly predictive test, the fraction of hits grows much more rapidly than that of false alarms, so the ROC curve shows a “knee” that approaches the upper left corner of the plot, and the area below the curve (AROC) tends to unit. This means that the statistical distributions of the test variable in the two populations, which the test aims to discriminate, are well separated, with little overlapping. A useless test, based on overlapping distributions, would have AROC = 0.5, a perfect test AROC = 1.

As repeated measures were performed in the same subject, mixed-effect linear regression models (nlme) (R 3.5.3) were studied to analyze the statistical association between the oxidative stress biomarkers and the metal concentrations. The model was of the type:

Lme (biomarker_conc(j)~ Sum (m_conc(i)) = ~1|subj)(1)

where the biomarker_conc (j) is the j-th outcome variable and the metal concentrations m_conc (i) are the predictors. The subjects were treated as a random variable and random intercepts were fitted.

The model fitting was iterated until the minimum Akaike Inference Criterion (AIC) and Bayesian Inference Criterion (BIC) indexes were obtained.

## 4. Results

The main characteristics of the studied subjects are reported in Table 1.

Based on the cotinine urinary levels, the result showed a higher frequency of smokers in the control group than in the welding workers’ group.

The ANOVA test for repeated measures relative to the concentrations of the 16 metals analyzed in this study gave a significant result (*p* = 0.001) for the three-level fixed effect “exposure” factor.

The concentrations of the 16 metals found in all the studied subjects are reported in Table 2, presented by their mean and standard deviation, median, 5° and 95° percentile, and number of analytical results below the limit of detection (LOD).

For the elements whose mean urinary concentrations were higher in workers than in controls or were higher at the end than before the working shift, the significance of the differences (*p*) has been shown using asterisks and circles.

For seven elements (Ba, Be, Bi, Cd, Hg, Fe, Pb), statistically significantly higher concentrations were found in the urine samples of the welders than in those of controls, both before and at the end of the working shift. Sr was higher than in controls only in urine samples collected before the shift, while the difference did not reach statistical significance at the end of the shift. Furthermore, Sr, Ni, and Ba significantly decreased at the end of the working shift.

From these results, it can be noted that these workers actually showed a very low occupational exposure to a variety of elements; only for eight of them was the effect of exposure on the dose biomarkers detectable: Ba, Be, Bi, Cd, Hg, Fe, Pb, Sr.

Exposure to Hg was also assessed by means of hair analysis. The mean value was 3.8 mg/kg of hair, but it was highly variable, between 0.025 and 16.61 mg/kg. The results were positively correlated to the urinary Hg concentration before the working shift (r = 0.47).

The repeated-measures ANOVA test relative to the concentrations of the biomarkers analyzed in this study gave a significant result (*p* = 0.009) for the three-level fixed effect factor “exposure”. Moreover, in this case, as in the test in which the metal concentrations were used as explanatory variables, a significant difference was found between exposed workers and control subjects.

The urinary concentrations of the five effect biomarkers, found in the same urine samples, both of the workers and of the controls, are reported in Table 3.

**Figure 1:**
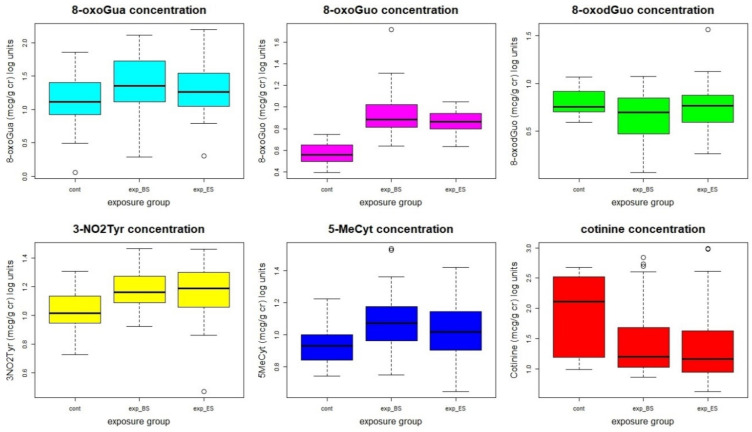
Statistical distribution of the oxidative stress biomarkers’ concentrations. The concentrations, originally in µg/g cr, were log-transformed to make the distributions more similar to a normal distribution.

No statistically significant differences were found between BS and ES values in workers. However, the mean values of 8-oxoGuo, 3-NO_2_Tyr, and 5-MeCyt were statistically significantly higher in workers than in controls, even if there was a higher percentage of smokers in the control group, showing the presence of an oxidative stress condition linked to the occupational exposure, probably to those elements that can accumulate in the body’s tissues. Figure 1 shows the statistical distribution of the concentrations of the oxidative stress biomarkers and of the cotinine.

**Figure 2:**
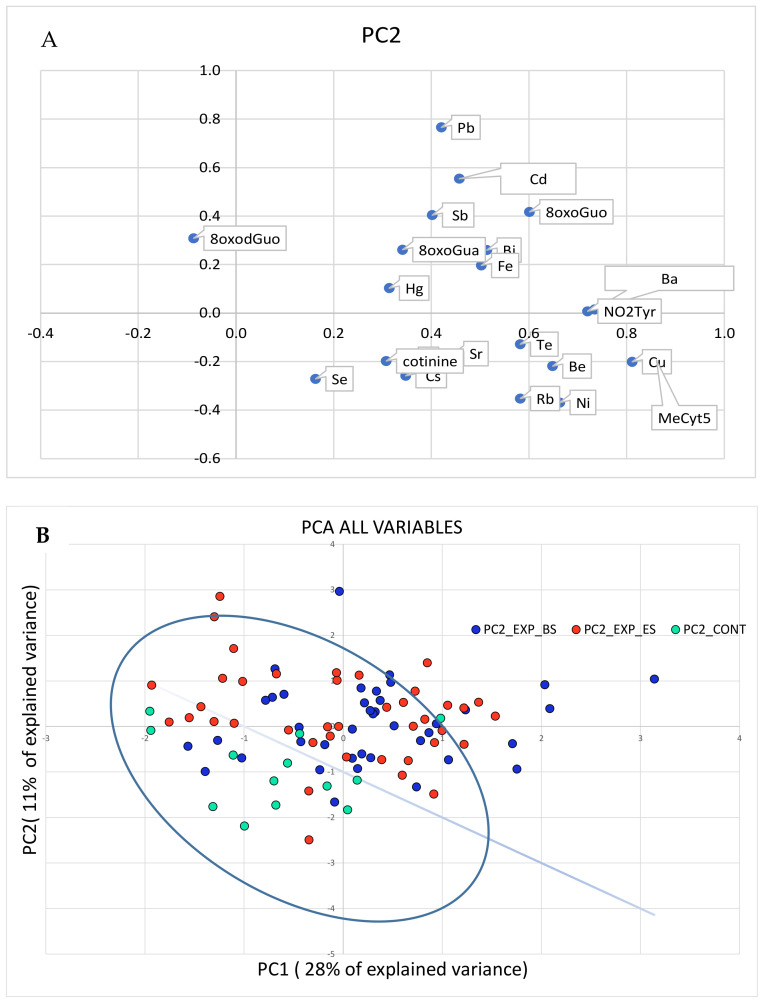
PCA analysis performed on all the variables, metals, and the biomarker concentrations. In panel (**A**), the original variables are represented in the rotated PC1-PC2 plane. The variables in the first quadrant are higher in the exposed than in the control group. In panel (**B**), the cases are plotted in the plane of rotated variables. The straight line approximately separates the exposed workers from the controls in the PC1-PC2 plane.

The results of the PCA performed with all the variables, the concentrations of metals, and the effect biomarkers are shown in Figure 2 (panels A and B).

As the PCA relative to the metal concentrations was not able to effectively discriminate the exposed subjects from the controls, the separation shown in Figure 1 can be mainly attributed to the biomarkers’ concentrations.

**Figure 3:**
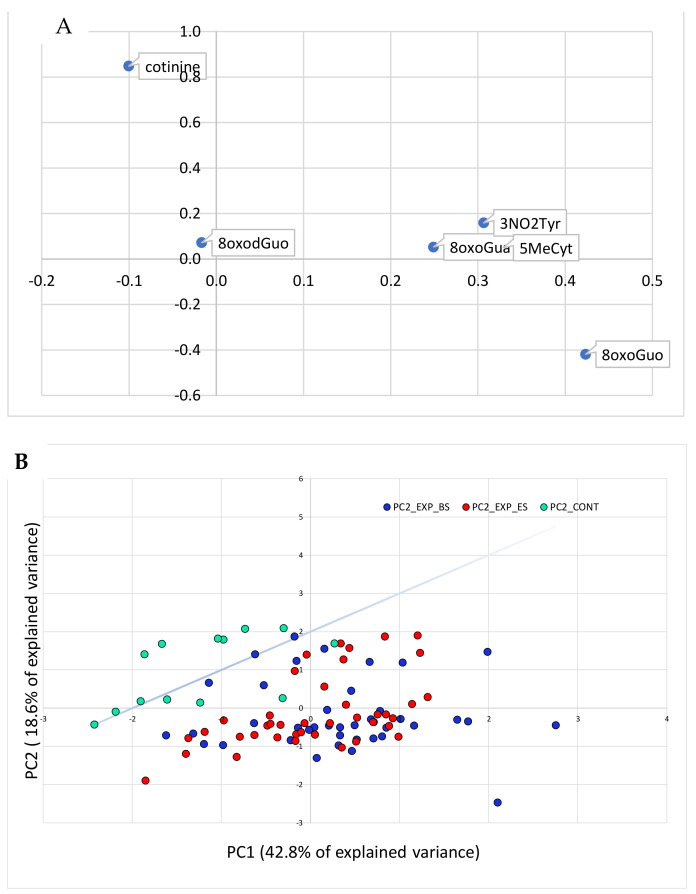
PCA analysis performed on the variables relative to the effect biomarkers and cotinine concentrations. In panel (**A**), the original variables are represented in the rotated PC1-PC2 plane. In panel (**B**), the cases are plotted in the plane of the rotated variables. The straight line approximately separates the exposed workers from the controls in the PC1-PC2 plane.

Figure 3 (panels A and B) shows the PCA relative to the effect biomarkers plus the cotinine concentrations.

In Figure 3, the separation between the exposed and the control groups in the space of the effect biomarker variables is even clearer than in Figure 2, in which also the exposure variables were taken into account.

It can be hypothesized that exposure to a low-dose heavy metal mixture produces a clear effect on the oxidative stress biomarkers and that these can be used as an early, sensitive, and specific diagnostic tool for the oxidative stress induced by the metals’ toxicity.

The ROC curve of the different biomarkers was studied to test this hypothesis.

**Figure 4:**
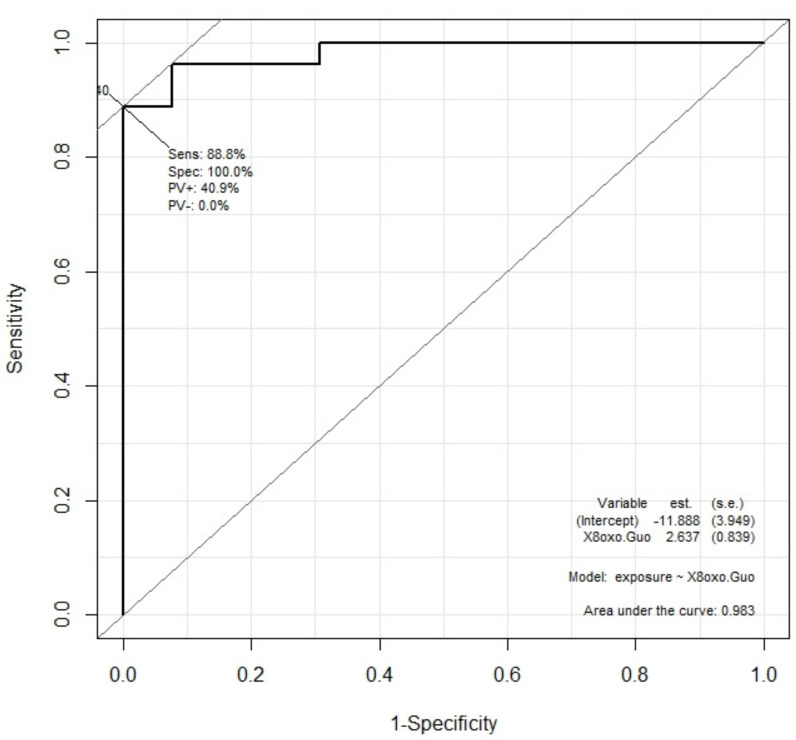
ROC curve for 8-oxoGuo concentration as diagnostic variable capable of dichotomic discrimination between exposed and control subjects. The area under the curve is 0.983.

The result is reported in the case of the 8-oxoGuo concentration in Figure 4. The area under the curve was larger than 0.98, showing the 8-oxoGuo concentration distribution for exposed and control subjects. A test based on the 8-oxoGuo concentration for discriminating exposed subjects from controls would potentially have high diagnostic power (very high sensitivity and specificity).

The area under the ROC curve was calculated for all five measured biomarkers and is reported in Table 4.

The result of the linear mixed-effect (lme) regression model fit is shown in Table 5. The biomarker concentrations are the outcomes and the heavy metal concentrations are the predictors. The β coefficients and their statistical significance are reported. As the parameters of the model were estimated by maximizing the likelihood, L, which can be increased by adding more parameters, to avoid overfitting, regularization parameters can be defined. Both the AIC and the BIC are parameters adding a penalty that increases with the number of model parameters. The lower the AIC and BIC, the more significant is the model.

As can be noted from Table 5, the fitting of mixed-effects multivariate linear models shows that the increase in the biomarkers’ concentrations, particularly 8-oxoGuo, 3-NO_2_Tyr, and 5-MeCyt, was significantly associated with an increase in the metals’ concentrations. Ba, Hg, Pb, and Sr were correlated with the RNA oxidative stress biomarker, 8-oxoGuo. Ba, Rb, Sr, Te, and V were correlated with the level of the protein oxidation biomarker 3-NO2Tyr, and Ba, Be, Cu, and Rb with 5-MeCyt, an epigenetic marker of RNA damage. In particular, all the biomarkers were associated with the Ba concentration. The 8-oxoGuo concentration was significantly associated with those metals whose concentration was significantly higher in exposed than in control subjects, whilst the biomarkers 3-NO_2_Tyr and 5-MeCyt were both associated with the metals with a similar concentration in exposed and control subjects.

## 5. Discussion

In this study, the biological monitoring of metal carpentry workers was carried out by measuring 16 urinary metal concentrations and five oxidative stress biomarkers, and the role of the occupational exposure to the metal mixture in determining an oxidative stress status was investigated, comparing the results to those of a control group working in the same company. Although the sample of control subjects was not very large, they worked for the same company and lived in the same geographical area, ensuring comparable environmental exposure to metallic elements.

It is important to remember that, for the purpose of occupational exposure assessment, not all elements were found to increase during the working shift, as most metals accumulate in the body tissues and are released over longer periods of time. Occupational exposure limits for some metals usually refer to pre-shift values, as they refer to the exposure of the previous days, or are indicated as non-critical, as they refer to the exposure of the past weeks or months. Table 6 summarizes the toxicokinetic properties of the eight elements for which a significant increase with respect to the control group was assessed, their reference values in the general population, and biological limits for occupational exposure, if existing.

Occupational exposure limits exist only for Hg, Cd, and Pb, but for Pb, they are based on the blood concentration. The ACGIH has set biological exposure limits for Hg at 20 µg/g of creatinine, sampling urine before the shift, for Cd at 5 µg/g of creatinine, sampling urine any time, and for Pb at [29]. The European Directive 983/2019, which was introduced in Italy in the D.Lgs 81/08 in February 2021, suggests a lower biological occupational exposure limit for Cd in the urine, equal to 2 µg/g of creatinine. The value set by the Italian law for Pb in blood is 600 µg/L blood, but it is reduced to 400 for women of childbearing age. The values found for Pb in blood during medical surveillance and those of Hg and Cd found in this study in these workers’ urine are well below the occupational biological exposure limits, confirming that a correct exposure assessment was carried out by the company. A limit of 2.3 μg/g for hair Hg was established by the Joint WHO/FAO Expert Committee on Food Additives for total Hg coming from food [34], which can be compared with the median value of 2.66 μg/g found by this study, showing that 50% of the workers’ hair was still below this limit.

The urinary levels of the metals determined in the urine of the workers, before and after the working shift, appear very low compared to the biological occupational exposure limits and comparable to the reference values found in the general population. Some of the values reported in Table 6 are expressed in µg/L, but, as creatininuria is accepted between 0.3 and 3 g/L, the order of magnitude is the same.

However, urinary concentrations of Ba, Be, Bi, Cd, Hg, Fe, Pb, and Sr were higher in welders than in controls and they were associated with higher values of 8-oxoGuo, 3-NO_2_Tyr, and 5-MeCyt, even in the presence of a higher percentage of smokers in the control group. This result shows the existence of an oxidative stress condition linked to the occupational exposure, probably to the mixture of those elements that can accumulate in the body’s tissues. It is interesting to note that all the biomarkers of effect studied are associated with the Ba urinary concentration, even if the adverse effects on human health of barium at low environmental doses are unknown.

In particular, the ROC curve of the 8-oxoGuo concentration for discriminating exposed subjects from controls shows very strong sensitivity and high specificity, confirming that this biomarker is the most sensitive to low-dose exposure to dangerous chemicals [7].

Workers exposed to carcinogenic and mutagenic substances in Italy undergo periodical medical surveillance, and these workers did not show any pathological signs. It is important to remember that the objective of studying early biomarkers is to provide a warning well before any symptoms appear.

This study presents some limitations. First, this was a cross-sectional study; thus, it did not permit any longitudinal evaluation. Second, the number of participants was limited, but this is often the case in Italy, where most workplaces are small–medium enterprises. Third, no dietary information was collected during the study, whereas food intake could be an important route of exposure for many of the studied elements. Lastly, the medical surveillance was not performed on the same day as the exposure assessment, so it was not possible to obtain information about the occupational exposure of each worker during the day of urine sampling and on previous days. However, as explained in the Discussion, it is important to consider that most metals accumulate in the body’s tissues and are released over longer periods of time, so the urine concentration does not refer to the exposure on the day of urine sampling.

## 6. Conclusions

This study presents the determination of urinary oxidative stress biomarkers associated with the urinary concentrations of several elements in metal carpentry workers, showing that the exposure/dose biomarkers of Ba, Be, Bi, Cd, Hg, Fe, Pb, and Sr are higher in workers than in controls, and they are associated with higher values of the effect biomarkers 8-oxoGuo, 3-NO_2_Tyr, and 5-MeCyt, measured in the same urine samples.

All the considered metals are known to have negative health effects, which can be reversible if early detected. Medical surveillance in the European Legislation is requested only for exposure to ascertained carcinogenic or mutagenic substances. The effect biomarkers’ determination could be used as a periodical test on workers exposed to dangerous substances as an early predictor of still-reversible adverse health effects. In particular, this study demonstrated that urinary 8-oxoGuo is a diagnostic marker of occupational exposure to metals. Its concentration could be monitored regularly in the exposed workers to detect any possible increase, in order to prevent the onset of occupational diseases.

Two important considerations emerge from these results.

Firstly, in occupational exposure assessment, it is very important to refer to local control subjects rather than referring to literature values, as biomarker levels can be increased even if very low, if the background is lower. The objective is to highlight the additional risk to which the workers are exposed, in order to prevent the future development of occupational diseases.

The second consideration is the importance of having a tool that helps to identify those workers that can be defined as “occupationally exposed” to chemicals, to whom the appropriate prevention measures and medical surveillance must be applied. It was demonstrated in this study that a test based on the 8-oxoGuo concentration for discriminating exposed subjects from controls has very high sensitivity without losing specificity (an area under the ROC curve larger than 0.98 was found). Therefore, the determination of urinary 8-oxoGuo appears to provide valuable support in identifying occupationally exposed workers.

In fact, as environmental monitoring measures the potential exposure of the workers to the considered pollutants, and biological monitoring of dose biomarkers (the elements in this case) measures the absorbed dose of the single substances, only the effect biomarkers can provide information about the impact that these doses have on workers’ health and about the additive or even synergistic effect that can be generated when they occur in a mixture.

## Figures and Tables

**Table 1:** Characteristics of the subjects.

Subjects	n.	Age (Years)	Smokers
Workers	40	Mean 53.4 range 29–65	9
Controls	13	Mean 44.9 range 20–59	7

**Table 2:** Urinary concentrations of metals in workers and controls (µg/g of creatinine).

**Before Shift (BS)**																
	**Hg**	**Be**	**Ni**	**Cu**	**Rb**	**Sr**	**Cd**	**Sb**	**Te**	**Cs**	**Ba**	**Pb**	**Bi**	**V**	**Fe**	**Se**
Mean	0.92 **	0.019 ***	4.9	23.7	913	157 *	0.61 **	0.17	1.67	7.7	11 ***	22 ***	0.07 **	0.68	9.1 *	37
std	0.91	0.020	1.8	9.7	383	69	0.40	0.15	0.92	3.8	11	42	0.10	0.98	8.0	10
Median	0.63	0.010	4.5	21.3	848	152	0.50	0.13	1.49	6.9	8	12	0.05	0.21	6.2	33
5° perc.	0.10	0.008	3.1	14.4	480	72	0.15	0.09	0.64	4.1	3	5	0.01	0.13	2.3	24
95° perc.	2.69	0.039	7.4	38.5	1650	252	1.30	0.26	3.25	13.6	34	46	0.17	2.74	25.1	52
% data >LOD	98	15	100	100	100	100	98	100	100	100	100	100	100	33	100	100
* *p* < 0.05; ** *p* < 0.01; *** *p* < 0.001 (BS higher than controls)																
**End of Shift (ES)**																
	**Hg**	**Be**	**Ni**	**Cu**	**Rb**	**Sr**	**Cd**	**Sb**	**Te**	**Cs**	**Ba**	**Pb**	**Bi**	**V**	**Fe**	**Se**
Mean	0.75 **	0.014 *	4.3 *°*	21.5	999	112 *°°°*	0.68 *	0.21	1.73	8.5	7.2 ***°	22 ***	0.07 *	1.0	9.3 *	42
std	0.62	0.010	2.2	8.5	392	48	0.45	0.17	0.71	4.5	3.5	32	0.09	1.3	7.2	17
Median	0.55	0.010	4.0	19.3	975	113	0.59	0.18	1.59	8.3	6.5	16	0.04	0.3	7.8	38
5°perc.	0.06	0.006	1.5	11.8	451	41	0.19	0.06	0.75	3.8	2.7	5	0.01	0.1	2.6	22
95° perc.	1.95	0.020	7.4	34.3	1700	182	1.63	0.50	2.86	15.6	12.4	39	0.16	3.5	21.2	70
% data >LOD	98	10	100	100	100	100	100	100	100	100	100	100	100	50	100	100
° *p* < 0.05; °°° *p* < 0.001 (ES significantly different from BS)																
* *p* < 0.05; ** *p* < 0.01; *** *p* < 0.001 (ES higher than controls)																
**Control Group**																
	**Hg**	**Be**	**Ni**	**Cu**	**Rb**	**Sr**	**Cd**	**Sb**	**Te**	**Cs**	**Ba**	**Pb**	**Bi**	**V**	**Fe**	**Se**
Mean	0.21	0.010 *	4.4	20.0	819	114	0.38	0.14	1.36	10	3.8	7.0	0.03	0.60	6.1	35
std	0.25	0.003 *	1.4	7.3	136	64	0.21	0.08	0.36	16	2.2	4.1	0.02	0.61	8.6	11
Median	0.09	0.010 *	4.1	18.3	795	84	0.29	0.13	1.36	5	3.1	5.4	0.02	0.26	3.1	34
5° perc.	0.02	0.007 *	3.0	12.9	642	46	0.17	0.06	0.83	4	1.7	2.6	0.01	0.12	2.4	17
95° perc.	0.74	0.015 *	6.7	31.5	1030	224	0.78	0.26	1.91	31	8.1	14.7	0.08	1.72	20.1	50
% data >LOD	92	0	100	100	100	100	100	100	100	100	100	100	100	46	100	100
* data calculated on the basis of LOD/2 to be able to be compared to the workers’ results																

**Table 3:** Urinary concentrations of biomarkers of effect biomarkers (µg/g of creatinine).

	8-oxoGua			8-oxoGuo			8-oxodGuo			3-NO2Tyr			5-MeCyt		
	C	BS	ES	C	BS	ES	C	BS	ES	C	BS	ES	C	BS	E
**Mean**	**20.12**	**34.62**	**26.87**	**3.76**	9.52 ***	7.56 ***	**6.66**	**5.04**	**6.68**	**11.33**	15.90 **	16.10 *	**9.05**	12.97 **	**11.60**
**std**	19.74	32.34	28.33	0.88	7.52	1.76	2.49	2.75	5.53	4.16	5.25	6.21	3.14	6.09	4.99
**Median**	13.13	22.51	18.29	3.60	7.67	7.33	5.71	4.96	5.85	10.32	14.52	15.51	8.51	11.84	10.39
5° perc.	2.32	4.01	6.67	2.66	4.87	4.98	4.37	1.22	2.46	6.16	9.22	8.45	5.59	6.33	5.65
95° perc.	52.13	101.37	65.50	5.10	13.15	10.49	11.34	10.12	11.98	18.36	25.52	27.05	14.08	23.50	21.43

* *p* < 0.05; ** *p* < 0.01; *** *p* < 0.001 (Higher than control group). All data are > LOD. C: control group. BS: before shift. ES: end of shift.

**Table 4:** ROC curves for the different biomarkers.

Variable (µg/g Creatinine) Log Units	Area	Standard Error	Significance	CL 95%	
				Lower Limit	Upper Limit
8-oxoGua	0.624	0.085	0.153	0.456	0.792
8-oxoGuo	0.983	0.012	0.000	0.960	1.000
8-oxodGuo	0.387	0.072	0.191	0.246	0.527
3-NO_2_Tyr	0.753	0.071	0.004	0.614	0.892
5-MeCyt	0.712	0.074	0.015	0.566	0.857

**Table 5:** Results of the fitting of the linear mixed-effect multivariate regressions.

(Dependent Variable 8-oxoGuo Conc. (µg/g Creatinine) Log Units			Dependent Variable 3-NO_2_Tyr Conc. (µg/g Creatinine) Log Units			Dependent Variable 5-MeCyt Conc. (µg/g Creatinine) Log Units		
Predictor (mcg/g Cr) Log Units	β Coeff. Adim	St. Error	Predictor (mcg/g Cr) Log Units	β Coeff. Adim	St. Error	Predictor (mcg/g Cr) Log Units	β Coeff. Adim	St. Error
Ba	0.231 ***	0.050	Ba	0.126 **	0.044	Ba	0.165 ***	0.041
Hg	0.057 **	0.028	Rb	0.268 **	0.075	Be	0.153 **	0.05
Pb	0.167 ***	0.046	Sr	0.217 ***	0.049	Cu	0.394 ***	0.082
Sr	0.134 *	0.064	Te	0.133 *	0.065	Rb	0.280 ***	0.070
			V	0.065 **	0.02			
Observations 93 Log Likelihood 48.800 Akaike Inf. Crit. −83.600 Bayesian Inf. Crit. −66.258			Observations 93 Log Likelihood 58.369 Akaike Inf. Crit. −100.738 Bayesian Inf. Crit. −81.011			Observations 93 Log Likelihood 81.650 Akaike Inf. Crit. −149.300 Bayesian Inf. Crit. −131.959		

Note: * *p* < 0.05; ** *p* < 0.01; *** *p* < 0.001.

**Table 6:** Information relevant to increased elements: Ba, Be, Bi, Cd, Fe, Hg, Pb, Sr.

Element	Half-Life in Urine	Reference Values in Urine µg/L	Biological Exposure Limit	Highest Mean Value in Welders in this Study	References
Ba	6 days ^a^	0.2–5 ^b^	-	11.39 µg/g creat. BS	^a^ [25] ^b^ [26]
Be	1–60 days ^c^	0.01–0.04 ^b^	-	0.019 µg/g creat. BS	^b^ [26] ^c^ [27]
Bi	15 days ^d^	0.8–1–6 ^c^	-	0.07 µg/g creat. BS and ES	^d^ [28] ^c^ [27]
Cd	7 h ^c^	0.1–1.5 ^b^	2 µg/g creatinine (EU) 5 µg/g creatinine (ACGIH [29])	0.068 µg/g creat. ES	^b^ [26] ^c^ [27]
Fe	no physiological excretion mechanism	up to 62.4 ± 4.1 µg/g creatinine in healthy subjects ^e^	-	9.25 µg/g creat. BS	^e^ [30]
Hg	1–3 months ^c^	1 ^c^	20 µg elemental Hg/g of creatinine (ACGIH [29])	0.092 µg/g creat. BS	^c^ [27]
Pb	1–2 months (in blood) ^c^; urine concentration reflects blood levels	12–27 ^c^	600–400 µg/L in blood 200 µg/L of blood (ACGIH [29]	22 µg/g creat. BS	^c^ [27]
Sr	0–6 days ^f^	40.9–505.8 ^g^ 80–350 ^h^	-	157.43 µg/g creat. BS	^f^ [31] ^g^ [32] ^h^ [33]

Letters a–h connect values to the appropriate reference in the table.

## Data Availability

The data presented in this study are available within this article. Further inquiries may be directed to the authors.
